# Recessive Mutations in *SPTBN2* Implicate β-III Spectrin in Both Cognitive and Motor Development

**DOI:** 10.1371/journal.pgen.1003074

**Published:** 2012-12-06

**Authors:** Stefano Lise, Yvonne Clarkson, Emma Perkins, Alexandra Kwasniewska, Elham Sadighi Akha, Ricardo Parolin Schnekenberg, Daumante Suminaite, Jilly Hope, Ian Baker, Lorna Gregory, Angie Green, Chris Allan, Sarah Lamble, Sandeep Jayawant, Gerardine Quaghebeur, M. Zameel Cader, Sarah Hughes, Richard J. E. Armstrong, Alexander Kanapin, Andrew Rimmer, Gerton Lunter, Iain Mathieson, Jean-Baptiste Cazier, David Buck, Jenny C. Taylor, David Bentley, Gilean McVean, Peter Donnelly, Samantha J. L. Knight, Mandy Jackson, Jiannis Ragoussis, Andrea H. Németh

**Affiliations:** 1 Wellcome Trust Centre for Human Genetics, University of Oxford, Oxford, United Kingdom; 2 NIHR Biomedical Research Centre Oxford, Oxford, United Kingdom; 3 Centre for Integrative Physiology, Euan MacDonald Centre for Motor Neurone Disease Research, University of Edinburgh, Edinburgh, United Kingdom; 4 Nuffield Department of Clinical Neurosciences, University of Oxford, Oxford, United Kingdom; 5 School of Medicine, Universidade Positivo, Curitiba, Brazil; 6 Russell Cairns Unit, Oxford University Hospitals NHS Trust, Oxford, United Kingdom; 7 Department of Paediatrics, Oxford University Hospitals NHS Trust, Oxford, United Kingdom; 8 Department of Neuroradiology, Oxford University Hospitals NHS Trust, Oxford, United Kingdom; 9 Department of Anatomy, Physiology, and Genetics, University of Oxford, Oxford, United Kingdom; 10 Royal Berkshire Foundation Trust Hospital, Reading, United Kingdom; 11 Illumina Cambridge, Saffron Walden, United Kingdom; 12 Department of Clinical Genetics, Oxford University Hospitals NHS Trust, Oxford, United Kingdom; University of Minnesota, United States of America

## Abstract

β-III spectrin is present in the brain and is known to be important in the function of the cerebellum. Heterozygous mutations in *SPTBN2*, the gene encoding β-III spectrin, cause Spinocerebellar Ataxia Type 5 (SCA5), an adult-onset, slowly progressive, autosomal-dominant pure cerebellar ataxia. SCA5 is sometimes known as “Lincoln ataxia,” because the largest known family is descended from relatives of the United States President Abraham Lincoln. Using targeted capture and next-generation sequencing, we identified a homozygous stop codon in *SPTBN2* in a consanguineous family in which childhood developmental ataxia co-segregates with cognitive impairment. The cognitive impairment could result from mutations in a second gene, but further analysis using whole-genome sequencing combined with SNP array analysis did not reveal any evidence of other mutations. We also examined a mouse knockout of β-III spectrin in which ataxia and progressive degeneration of cerebellar Purkinje cells has been previously reported and found morphological abnormalities in neurons from prefrontal cortex and deficits in object recognition tasks, consistent with the human cognitive phenotype. These data provide the first evidence that β-III spectrin plays an important role in cortical brain development and cognition, in addition to its function in the cerebellum; and we conclude that cognitive impairment is an integral part of this novel recessive ataxic syndrome, Spectrin-associated Autosomal Recessive Cerebellar Ataxia type 1 (SPARCA1). In addition, the identification of SPARCA1 and normal heterozygous carriers of the stop codon in *SPTBN2* provides insights into the mechanism of molecular dominance in SCA5 and demonstrates that the cell-specific repertoire of spectrin subunits underlies a novel group of disorders, the neuronal spectrinopathies, which includes SCA5, SPARCA1, and a form of West syndrome.

## Author Summary

β-III spectrin is present in the brain and is known to be important in the function of the cerebellum. Mutations in β-III spectrin cause spinocerebellar ataxia type 5 (SCA5), sometimes called Lincoln ataxia because it was first described in the relatives of United States President Abraham Lincoln. This is generally an adult-onset progressive cerebellar disorder. Recessive mutations have not previously been described in any of the brain spectrins. We identified a homozygous mutation in *SPTBN2*, which causes a more severe disorder than SCA5, with a developmental cerebellar ataxia, which is present from childhood; in addition there is marked cognitive impairment. We call this novel condition SPARCA1 (Spectrin-associated Autosomal Recessive Cerebellar Ataxia type 1). This condition could be caused by two separate gene mutations; but we show, using a combination of genome-wide mapping, whole-genome sequencing, and detailed behavioural and neuropathological analysis of a β-III spectrin mouse knockout, that both the ataxia and cognitive impairment are caused by the recessive mutations in β-III spectrin. SPARCA1 is one of a family of neuronal spectrinopathies and illustrates the importance of spectrins in brain development and function.

## Introduction

Spectrins are a diverse family of membrane scaffold proteins. They were originally found in erythrocytes where mutations result in various haemolytic anemias [1], [2]. Spectrins have been identified in the brain [3] but until recently little was known of the effects in humans of brain spectrin mutations. In 2006, heterozygous mutations of the brain spectrin gene *SPTBN2*, encoding β-III spectrin, were found to cause Spinocerebellar Ataxia Type 5 (SCA5) [4]. SCA5 is an autosomal dominant, slowly progressive, adult onset, pure cerebellar ataxia, which was first identified in a large family who are the descendents of relatives of the US President Abraham Lincoln; SCA5 is therefore sometimes referred to as “Lincoln ataxia” [5], [6], [7]. Two other SCA5 families have been described in the literature, one from France and one from Germany [8], [9].

β-III spectrin is a 2,390 amino acid protein comprising an N terminal domain containing the actin/ARP1 binding site, 17 spectrin repeats, (the latter containing regions which bind the glutamate transporter EAAT4 [10], and ankyrin [11]), and a C terminal domain of uncertain function. β-III spectrin forms antiparallel tetrameric heterodimers with α-II spectrin, encoded by *SPTAN1*. The tetrameric self-association probably requires the presence of the C terminal β spectrin repeats, B16 and B17, and the N terminal α spectrin repeats, A0 and A1, with absence of these regions highly likely to impair the formation of a functional tetramer [12]. Three heterozygous dominant mutations in *SPTBN2* have been reported to cause SCA5: in the US (Lincoln) family a 13 amino acid in-frame deletion (E532_M544del) in the third spectrin repeat, in the French family a small complex in-frame deletion-insertion (L629_R634delinsW), also in the third spectrin repeat, and in the German family a missense mutation (L253P), in the N terminal domain. The mechanism of action of these mutations is not immediately obvious and could be explained by haploinsufficiency, in which the mutant allele is inactive and the normal stoichiometry for tetramer formation is lost, a dominant negative effect which suppresses wild type (wt) function, or a gain of function effect. Several lines of evidence have suggested that a dominant negative effect in SCA5 is most likely. Using targeted gene disruption of mouse β-III spectrin, Perkins et al, reported that homozygous knockout mice (β-III spectrin −/−) had cerebellar ataxia, a progressive loss of cerebellar Purkinje cells and an associated decrease in the Purkinje cell specific glutamate transporter EAAT4 [13]. The β-III spectrin −/− mutant mice lack all full-length β-III spectrin but do express, at a low level, a form of β-III spectrin (∼250 KDa) that lacks most of the actin-binding domain encoded by exons 2–6. The heterozygous mice (β-III spectrin +/−) were reported to be normal. Further work has shown that the L253P (German) missense mutation has a dominant negative effect on wild type function by preventing protein trafficking from the Golgi apparatus [14]. There is evidence also that *de novo* in-frame mutations in *SPTAN1* encoding α-II spectrin have dominant negative effects, causing a form of West Syndrome (infantile epilepsy with developmental delay) [15]. However, although experimental data has strongly suggested that small in-frame mutations or missense mutations in α-II or β-III spectrins have a dominant negative effect, no recessive mutations in spectrins have been found, and such data would lend further strong support for this hypothesis.

Here we report the first description of recessive mutations in *SPTBN2* in which there is a severe developmental childhood ataxia but also significant cognitive impairment. The homozygous stop codon c.1881C>A (p.C627X), was identified in three affected individuals from a consanguineous family using targeted capture and next generation sequencing and both the ataxia and cognitive impairment co-segregate with the mutation. However, since more than one mutation can co-segregate, particularly in consanguineous families, we considered whether a second recessive mutation, either homozygous or compound heterozygous, could account for the cognitive impairment. We investigated this using a combination of SNP array analysis and whole genome sequencing, but found no evidence of a second mutation.

We also investigated β-III spectrin *−/−* knockout mice [13] for supportive evidence that the cognitive impairment in the human subjects is caused by loss of β-III spectrin. We examined the mouse model for morphological abnormalities of neurons in brain regions (other than cerebellum), which are thought to be involved in memory function including prefrontal cortical (PFC) layers, the caudate putamen/striatum and hippocampus (HPC). Finally we tested the mice using object recognition tasks, which have been shown to correlate with function of the PFC and HPC [16], [17]. The morphological and behavioural abnormalities found in the knockout mice provide further evidence that the cognitive impairment in our human subjects is an integral part of this novel recessive disorder which we have called SPARCA1 (“Spectrin-associated Autosomal Recessive Cerebellar Ataxia type 1”). We suggest that this represents one of a novel group of disorders, the neuronal spectrinopathies, which demonstrate that the cell-specific functional repertoire of spectrin subunits are involved in brain development including the cortex, in addition to cerebellar development and function.

## Results

### Clinical phenotype and genetic analysis

**Figure 1:**
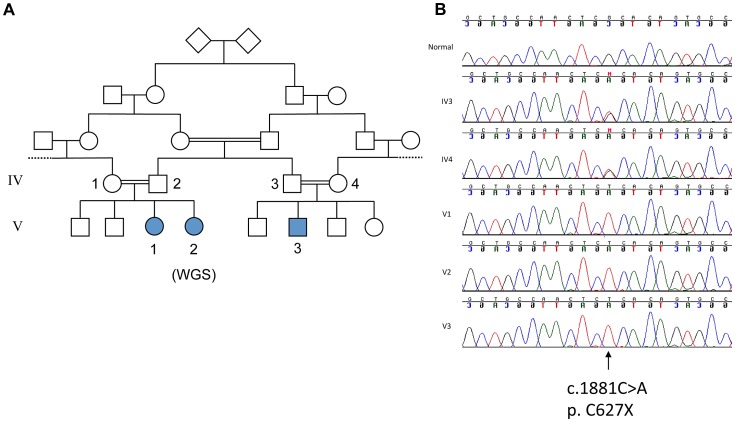
Genetic analysis of family with ataxia and cognitive impairment. A. Pedigree of family. B. Sanger sequencing of the mutation c. 1881C>A; p.C627X in normal, parents of V3 (IV3 and IV4) and affecteds, V1, V2, V3.

**Figure 2:**
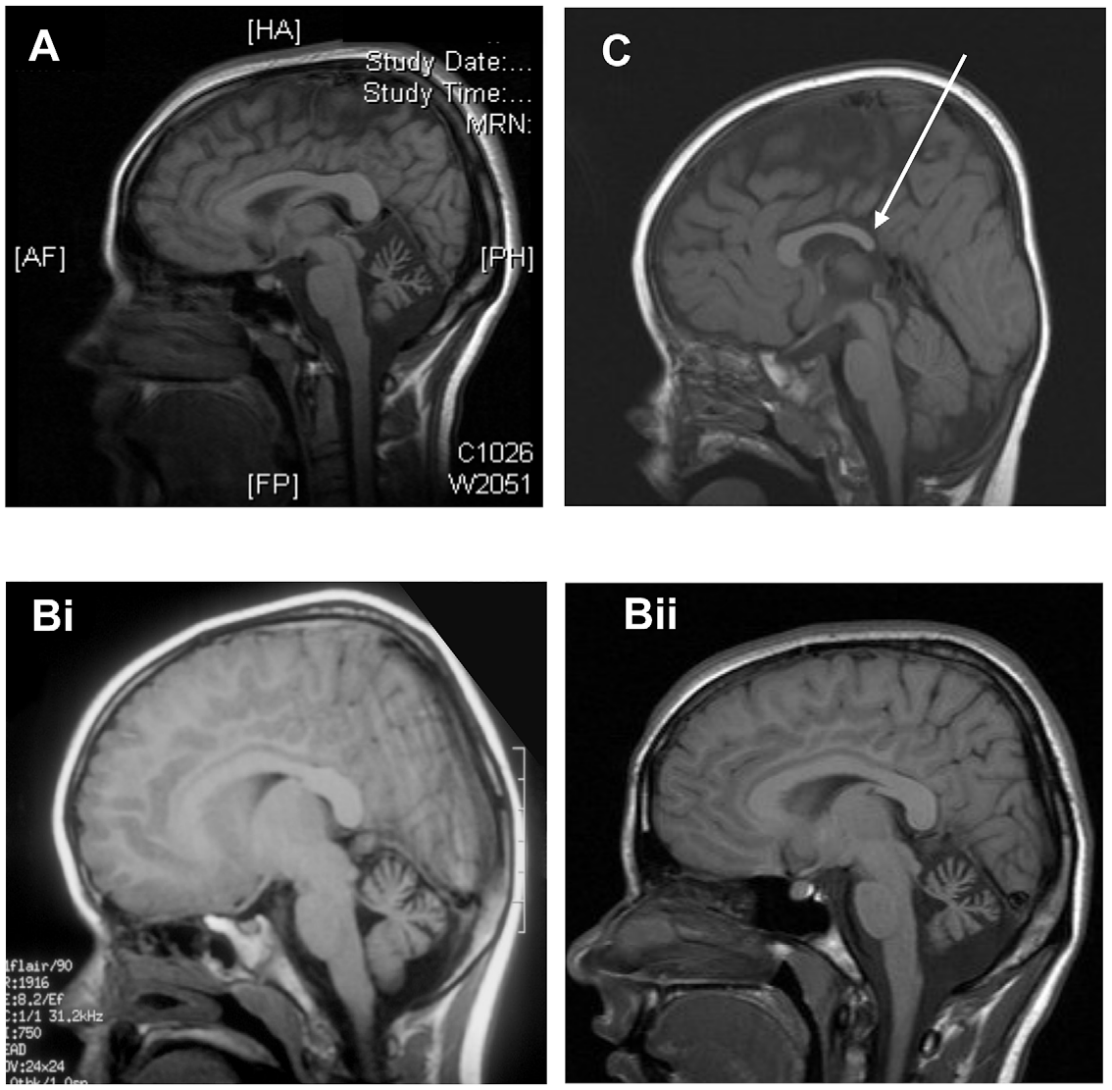
Neuroimaging of patients. A. Sagittal T1w MRI in subject V1 age 21 demonstrating clear cerebellar atrophy. B. Sagittal T1w MRI in subject V2 at age 6. Sagittal T1w MRI in subject V2 age 16 shows clear atrophy of the cerebellum. C. Sagittal T1w MRI in subject V3 showing hypoplasia of posterior corpus callosum (white arrow).

The three affected individuals are from a UK family of Pakistani origin with complex consanguinity (see Figure 1A), but no other family history of neurological disorders. The clinical phenotype in the 3 individuals is identical (Table 1). V1 was referred at the age of 13 months with motor delay; she was extremely floppy and was unable to crawl. She sat at 10 months, crawled at 18 months and was pulling to stand at 20 months. She walked with a walker by the age of 5 and started to walk with support at age 7. She was noted to have language delay and at age 5 was just starting to join words together. Global developmental delay was subsequently noted, she was educated at a special school and now attends a college for adults with special educational needs. On examination there are abnormal eye movements with a convergent squint, hypometric saccades, jerky pursuit movements, and an incomplete range of movement particularly in the horizontal plane. There is obvious dysmetria and dysdiadochokinesia of the limbs and gait ataxia with inability to tandem walk without falling. Limb tone is normal, reflexes are normal and plantars flexor and there is no evidence of any sensory abnormality. Rombergs sign is normal. Neuropsychological assessment reveals significant global cognitive impairment with all IQ scales falling at the second percentile or below, and with Full Scale IQ scores falling in the learning disabled range (Table 1). A brain CT scan at age 2 did not show any abnormality, but a recent MRI brain reveals significant cerebellar atrophy (Figure 2A). V2 is the younger sibling of V1. She was noted to have developmental delay in early childhood and also did not start to walk until age 7. On examination, she has an identical clinical phenotype to that of her sister except for occasional beats of nystagmus on eye examination. She attends a school for children with learning disabilities and a recent assessment (at age 16) shows functioning in English and Mathematics at the level of an average 5–7 year old in the UK requiring special educational support. Formal cognitive assessment also showed very similar impairments to V1 with scores on all IQ scales falling at the second percentile or below, and with Full Scale IQ scores falling in the learning disabled range (Table 1). The difference between Verbal and Performance IQ for each individual was not statistically significant (p = 0.15). MRI imaging in V2 at age 6 revealed cerebellar atrophy and this was found to have progressed over time (Figure 2Bi and Bii). V3 is the first cousin of V1 and V2. He was noted to have poor head control and balance in early childhood. Clinical examination is identical to his cousins and also shows an identical developmental profile in that he has just started to walk with assistance at the age of 7. He also has an identical eye movement disorder, a convergent squint, dysmetria and dysdiadochokinesia. He is hypotonic with normal reflexes downgoing plantars and no evidence of a sensory neuropathy. He attends a mainstream school but requires full time one to one support. Cognitive assessment of V3 also showed significant global cognitive impairment (Table 1). The slightly higher IQ scores in V3 results from a floor effect in the normative data rather than a significant difference in cognitive ability from his older cousins. In this age cohort the lowest attainable scores are VIQ = 62, PIQ = 73 and FSIQ = 63 and therefore V3 falls in the same learning disabled range as his cousins. Brain imaging of V3 showed a normal cerebellum at age 5, but mild hypoplasia of the posterior corpus callosum (Figure 2C). The normal appearance of the cerebellum in V3 at an early age is not unexpected as both his cousins imaging shows progression with time. Neurological examination of both sets of parents was entirely normal, with no evidence of ataxia. The father of V1 and V2 works as a bus driver, having left school at age 16 with 5 GCSEs (General Certificates of Secondary Education) and the father of V3 works in a warehouse and has a similar educational background. Formal psychometric testing in the father of V1 and V2 showed IQ indices falling in the low average range consistent with his educational attainment. The father of V3 was not available for testing but has very similar attainment levels to his brother. Formal assessment of the mothers could not be performed since neither speak English, but interview of the family did not reveal any evidence of learning disability. There is no history of the siblings or grandparents of the affected individuals having any cognitive or neurological abnormalities.

**Table 1:** Clinical and neuropsychological assessments of family.

		V1 (aged 21)	V2 (aged 16)	V3 (aged 7)	IV1 (Father of V1 and V2)	IV2 (Mother of V1 and V2)	IV3 (Father of V3)	IV4 (Mother of V3)
Neurological examination	Tone	N	N	N	N	N	N	N
	Power	N	N	N	N	N	N	N
	Co-ordination	Dysmetria and Dysdiadochokinesis	Dysmetria and Dysdiadochokinesis	Dysmetria and Dysdiadochokinesis	N	N	N	N
	Reflexes	Normal	Normal	Normal	N	N	N	N
	Sensation	Normal	Normal	Normal	N	N	N	N
	Gait	Unsteady, walks independently	Unsteady, walks independently	Unsteady, only walks with assistance	N	N	N	N
	Rombergs	negative	negative	negative	N	N	N	N
	Eye movements	Delayed saccadic initiation	Delayed saccadic initiation, first degree nystagmus in horizontal plane	Delayed saccadic initiation	N	N	N	N
MRI Brain		N/A	Cerebellar atrophy		N/A	N/A	N/A	N/A
IQ		48	48	?				
Educational assessment		Special School	Special School	Full assistance at Mainstream School	Normal School	Normal School	Normal School	Normal School
IQ Scale	Index							
WASI	Verbal IQ (centile)	55 (0.1)	58 (0.3)	66 (1)	80 (9)	N/A	N/A	N/A
	Performance IQ (centile)	54 (0.1)	56 (0.2)	73 (4)	84 (14)	N/A	N/A	N/A
	Full Scale IQ (centile)	51 (0.1)	53 (0,1)	67 (1)	80 (9)	N/A	N/A	N/A

We initially performed targeted capture of >100 known ataxia genes (including *SPTBN2*) in a group of children with unexplained ataxia including patient V3, followed by next generation sequencing. In V3 we identified only one mutation, a homozygous stop codon p. C627X (c.1881C>A), located in the third spectrin repeat in *SPTBN2* and used Sanger sequencing to confirm that all three affected patients in the family had the same mutation whereas the neurologically normal parents of V3, were shown to be heterozygous for the mutation (Figure 1B). Since mutations in β-III spectrin are associated with cerebellar degeneration in SCA5, the newly identified mutation was considered likely to explain the ataxia, although of a developmental type with a much earlier onset. However, since more than one mutation can co-segregate, particularly in consanguineous families, we went on to consider the contribution of the mutation in *SPTBN2* to the observed cognitive impairment. We therefore used SNP array analysis and whole genome sequencing to search for any evidence of a second mutation.

### SNP array genotyping

**Figure 3:**
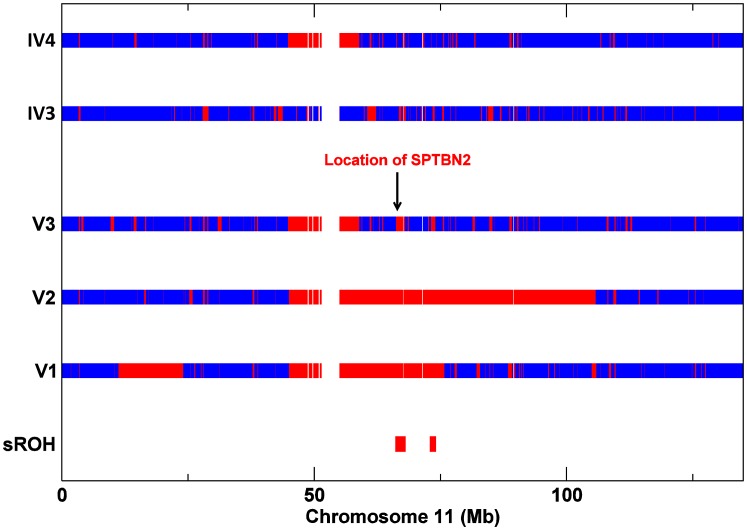
SNP Zygosity data for chromosome 11 from affecteds V1, V2, V3, and V3's parents IV3 and IV4. Red lines correspond to homozygous SNPs and blue lines to heterozygous SNPs (the gap represents the centromere).

To investigate whether a second homozygous mutation segregated with the cognitive impairment, all 3 affected individuals (V1, V2 and V3) and the unaffected parents of V3 (IV3 and IV4) were genotyped to identify regions of homozygosity (ROH) shared by V1, V2 and V3 and not present in either IV3 or IV4. This analysis identified 20 shared homozygous segments on autosomes totalling 17.1 Mb (Table 2). *SPTBN2*, on chromosome 11, was located in the largest ROH shared by V1, V2 and V3 and not present in either IV3 or IV4 (Figure 3).

**Table 2:** Regions of homozygosity in V1, V2, and V3.

ROH	ROH size (bp)	Comments
chr1:11,008,695–11,512,411	503,716	
chr1:152,466,882–152,773,905	307,023	
chr2:110,432,886–111,586,214	1,153,328	Contains *NPHP1* synonymous missense variant, L551L
chr4:1,755,491–2,268,126	512,635	
chr5:42,431,016–42,911,014	479,998	
chr6:42,231,419–43,196,182	964,763	
chr7:73,889,810–75,160,045	1,270,235	
chr8:92,965,409–93,493,424	528,015	
chr11:44,874,510–51,372,036	6,497,526	Homozygous in IV4
chr11:55,091,268–59,054,448	3,963,180	Homozygous in IV4
**chr11:66,108,660–68,097,826**	**1,989,166**	**Contains ** ***SPTBN2*** ** stop codon**, C627X
chr11:72,937,274–74,146,105	1,208,831	
chr12:825,782–1,583,962	758,180	
chr12:88,356,316–89,340,293	983,977	
chr14:105,777,094–106,863,833	1,086,739	
chr15:48,369,485–48,889,188	519,703	
chr16:47,239,089–48,179,983	940,894	
chr16:50,034,680–50,641,988	607,308	
chr16:61,713,393–63,225,217	1,511,824	
chr17:17,544,704–18,634,672	1,089,968	Homozygous in IV3
chr17:27,935,688–28,543,044	607,356	
chr17:39,993,771–41,059,014	1,065,243	
chr18:21,155,324–21,264,965	109,641	

### Whole-genome sequencing

Whole genome sequencing of patient V2 was performed on the Illumina HiSeq2000 as 100 bp paired end reads, using v3 clustering and sequencing chemistry. After duplicate reads removal, the mean coverage across the genome was 25.6× with 90.4% of bases covered at 15× or more. The mean coverage over the 17.1 Mb ROH identified by SNP analysis was 25.9× with 93.4% of bases covered at 15× or more. Variant calling was performed as detailed in the Materials and Methods.

We firstly based our data analysis on an autosomal recessive disease model, caused by one or more rare homozygous mutations and focused on homozygous variants occurring in the shared ROH identified by SNP array analysis, filtering them out if they were:

- present in 1000 Genomes with an allele frequency >1% (http://www.1000genomes.org/)
- in a region of segmental duplication
- observed as homozygous in other WGS500 samples within our Institute (see Materials and Methods)

These filtering steps identified 68 candidate variants, subdivided into functional classes (Table 3). Only 2 exonic variants were found: a synonymous variant, *NPHP1* L551L on chr2 which is not predicted to be pathogenic and is not located near a splice site, and the stop codon C627X in *SPTBN2* on chr11 (Table 2 and Table 3). Of the remaining variants, 21 were intergenic and also considered unlikely to be disease related, and 4 variants were in untranslated regions (5′ UTR) or in non-coding RNAs and all were in positions which scored poorly with PhyloP and GERP. In addition, none of the associated genes (*UBIAD1*, *LINC00116*, *LOC100130987*) appear to be relevant for this disorder. The other 41 were in intronic and upstream regions but based on evolutionary conservation and available information in databases (eg HGMD [18]) we found no evidence of potential involvement in the disease. The only likely pathogenic variant is the stop codon in *SPTBN2*.

**Table 3:** Number of candidate variants per functional class.

Functional Class	Number of variants
Exonic Total	2
• Stop Gained	1
• Synonymous	1
5′ UTR	1
ncRNA	3
Intronic	39
Upstream	2
Intergenic	21

We also considered a model of recessive inheritance with compound heterozygous mutations segregating with the ataxia and/or cognitive impairment. Our criteria were that all 3 affecteds must have two different variants in the same gene and where this occurred the variants should be *in trans* (*ie* each parent is a carrier). We identified all potential compound heterozygous coding variants present in the WGS data for individual V2. In total there were variants fulfilling our criteria at 13 different loci but in only 1 case were both variants present in all 3 affecteds and further analysis revealed that in this instance both variants were also in the father of V3 (*ie* were *in cis*). Furthermore, none of the variants identified are known to be associated with ataxia or cognitive impairment and the majority of genes had data suggesting an alternative function (such as taste or fertility), nor were there any likely candidates based on pathogenicity bioinformatic prediction programs (Table S2).

### Abnormal dendritic morphology of prefrontal cortical neurons in β-III spectrin −/− mice

**Figure 4:**
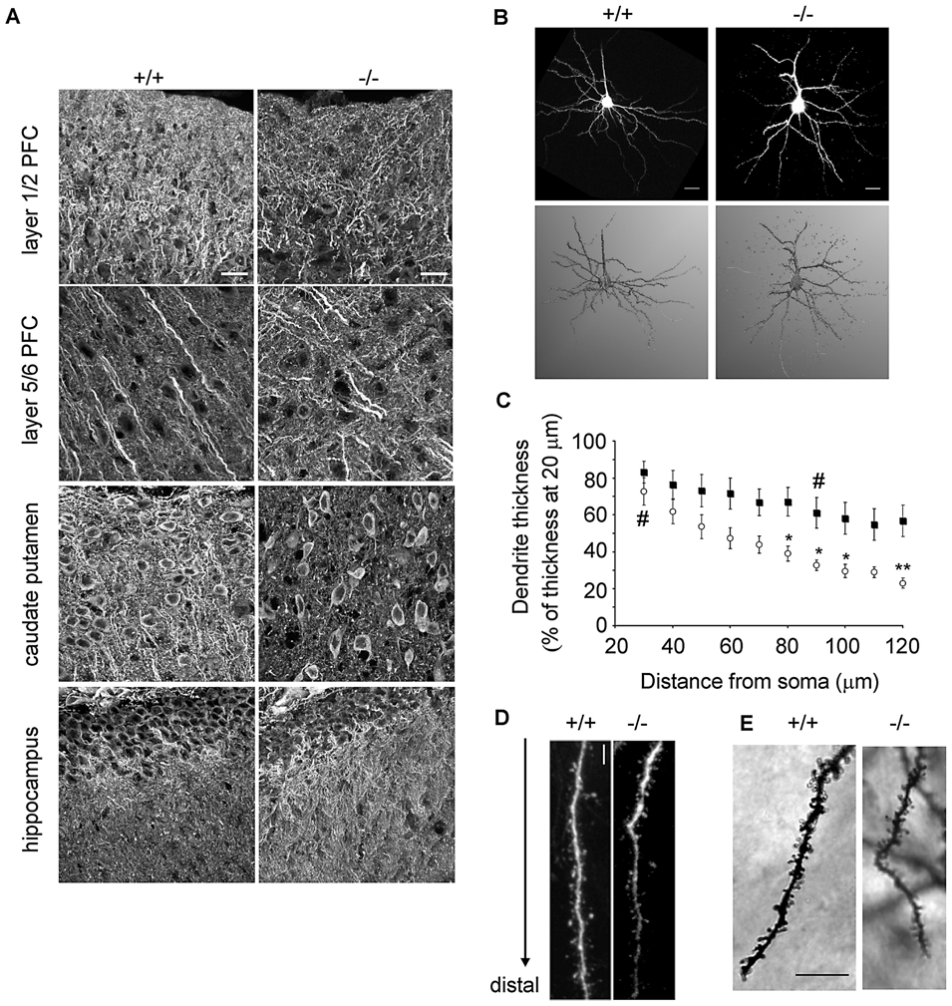
Abnormal dendritic morphology in β-III spectrin −/− mouse compared to wild type. A. Sagittal sections immunostained for MAP2 show irregular reactivity throughout prefrontal cortical layers and caudate putamen/striatum of 6-week-old β-III spectrin knockout (−/−) mice compared to wild type (+/+) but normal staining within hippocampus (N = 3 each genotype; Bar, 20 µm). B. Top, Representative images of pyramidal neurons in layer 2/3 prefrontal cortex from 8-week-old WT and β-III spectrin knockout mice filled with Alexa 568 (Bar, 20 µm). Bottom, Neuronal 3-D reconstruction over laid using NeuronStudio software. C. Quantification of basal dendrite morphological parameters measured from reconstructed images shows greater distal thinning of dendrites in cells from β-III spectrin knockout mice (open circles; N = 9) compared with WT cells (filled squares; N = 8). All data are mean ± SEM (* denotes significance between groups and # significance within a group.) D. High magnification image of single basal dendrite showing distal thinning in β-III spectrin knockout compared to WT but presence of normal spines (Bar, 5 µm). E. High magnification image of Golgi impregnated pyramidal neuron from prefrontal cortex of WT and β-III spectrin knockout mice (Bar, 10 µm).

The phenotype of our patients suggested that β-III spectrin is involved in cognitive development, in addition to being essential for motor functions. We therefore utilised β-III spectrin knockout mice which have progressive cerebellar degeneration and lack any full length β-III spectrin [13], to further investigate the role of β-III spectrin in other brain regions. Our previous work revealed that β-III spectrin is required for the correct dendritic development of Purkinje cells [19], [20] and therefore we initially examined dendritic organisation in other brain regions by immunostaining sagittal sections from the brains of 6-week-old wild-type and β-III spectrin knockout animals for microtubule associated protein 2 (MAP2), a dendritic marker. This revealed irregular reactivity throughout the PFC layers and within the caudate putamen/striatum of knockout animals when compared to WT mice but no obvious difference in the HPC (Figure 4A). However no difference was observed between WT and β-III spectrin knockout animals when the cortex and striatum were immunostained for tau or myelin basic protein (MBP) indicating that there was no change to axonal structure (Figure S1).

The PFC in humans is believed to be important for complex cognitive tasks, and given there is evidence of a close association between this area and the neocerebellum, as well as high expression levels of β-III spectrin in mouse [10] we further investigated the prefrontal cortical region in β-III spectrin knockout animals. There was no difference in the thickness of individual prefrontal cortical layers (data not shown) but the morphology of individual pyramidal neurons in β-III spectrin knockout animals was found to be altered. Morphometric analysis of dye-injected pyramidal neurons from layer 2/3 showed basal dendrites in 8-week-old β-III spectrin knockout mice were significantly thinner distally compared to wild type cells (Figure 4B–4D). Moreover, the basal dendrites of knockout mice tapered more rapidly than those of wild types, being significantly reduced in thickness between 20 and 30 µm from the soma, whereas wild type dendrites showed no significant narrowing until 90 µm from the soma. However, no difference in spine density was observed between genotypes in either dye injected (Figure 4D: +/+, 2.8±0.6, n = 8; −/−, 3.2±0.2 spine/µm^3^, n = 7; p = 0.56) or Golgi-impregnated (Figure 4E: +/+, 12.4±1.7, n = 4; −/−, 13.7±1.3 spine/10 µm, n = 6; p = 0.56) pyramidal neurons. Only small sections of apical dendrites could be reconstructed from the serial stacks of dye-injected cells. Nevertheless, quantification of the short regions imaged, when normalized to length analysed, indicated reduced apical dendritic volumes, and hence thinner apical dendrites in β-III spectrin knockout animals (+/+, 4.3±0.47; −/−, 2.5±0.36 µm^3^/µm, n = 6 for each genotype; p = 0.011).

### Corpus collosum appears normal in β-III spectrin −/− mice

**Figure 5:**
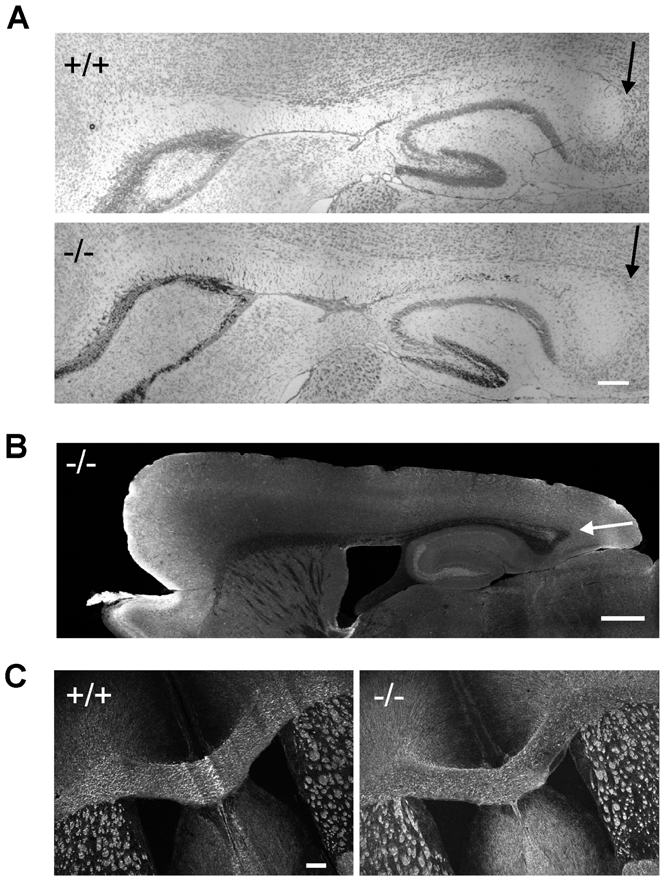
Absence of hypoplasia of posterior corpus callosum in β-III spectrin knockout mice. Sagittal sections from 8-week old WT (+/+) and knockout (−/−) animals stained with cresyl violet (A, Bar 200 µm) and anti-tau antibody (B, Bar 500 µm) with arrow pointing to posterior corpus callosum. C. Coronal sections immunostained for MBP (Bar 200 µm).

Since patient V3 shows mild hypoplasia of the posterior corpus callosum we examined this brain structure in 8-week old β-III spectrin knockout animals to determine if the morphological defect in the human subject could be a consequence of β-III spectrin loss or is unlinked to the homozygous stop codon c.1881C>A (p.C627X) mutation in *SPTBN2*. No signs of posterior hypoplasia were observed in sagittal sections stained either with cresyl violet (Figure 5A) or an anti-tau antibody (Figure 5B). Similarly width of corpus callosum, measured from coronal sections immunostained for MBP (Figure 5C), was no different between WT and knockout animals (+/+, 469.7±46.6; −/−, 480.6±41.3 µm, N = 3 for each genotype; p = 0.28).

### β-III spectrin −/− mice are deficient in behaviour tasks

**Figure 6:**
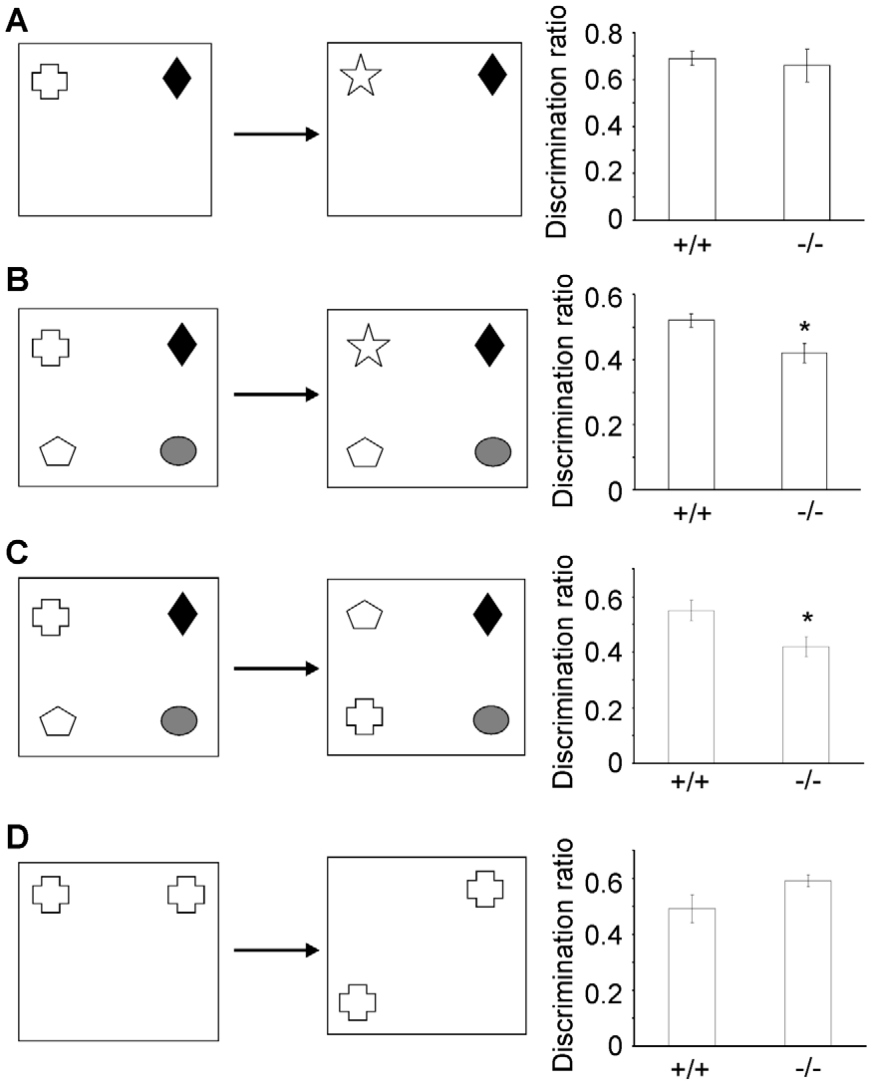
β-III spectrin knockout mice display deficits in some object recognition tasks. Diagram of task and performance of WT (+/+) and β-III spectrin knockout mice (−/−) in the four object recognition tasks. Two-novel object recognition (A), four-novel object recognition (B), object-in-place (C) and object location task (D). All data are mean ± SEM (N = 6–9; * *P*<0.05).

Four object recognition memory tasks (two- and four- novel object preference, object-in-place and object location; Figure 6A–6D) were carried out to assess whether β-III spectrin knockout animals displayed any cognitive deficits. No impairment in the two novel object recognition task (“object identity”) was observed in β-III spectrin knockout animals compared with wild type animals (Figure 6A); however knockout animals performed worse in the four novel object recognition task (Figure 6B). Knockout animals were also worse at discriminating between rearranged and non-rearranged objects in the object-in-place task compared with litter mate controls, shown by their failure to spend more time exploring the two objects in different locations compared with the two objects that had not moved (“object displacement”) (Figure 6C). However, there was no significant difference in performance for the object location task (Figure 6D). The poorer performance in the four-novel object recognition task for knockout animals was not a consequence of less exploration in the 5 minute sample phase as in fact they explored more than wild type animals (+/+, 64.9±6.7; −/−, 88.7±4.8 sec; p = 0.018). Similarly for the object-in-place task although there was no significant difference between genotypes there was a trend for greater exploration in knockout animals (+/+, 42±3.6; −/−, 62.2±8.7 sec; p = 0.054).

## Discussion

The integrated evidence from clinical, genetic and neuropsychological analysis in humans and behavioural and morphological analysis in a mouse model demonstrate that we have identified a novel recessive disorder, SPARCA1, associated with mutations in β-III spectrin. The 3 human subjects with a premature stop codon and the mouse knockout all have very early onset cerebellar ataxia, indicating a developmental role for β-III spectrin. The human and mouse knockout phenotype also show that β-III spectrin is involved in cognitive development and function. The human subjects have global cognitive impairment in the mild/moderate range. The specific brain structures and connections associated with this impairment are not yet known and further detailed neuropsychological testing will be required. However, we have shown that in the mouse knockout there are morphological abnormalities especially thinning of dendrites in PFC neurons, similar to that previously reported for Purkinje neurons [19], but with no obvious changes in various regions of HPC (CA1, CA3 and dentate gyrus), and the behavioural tests in the mouse are consistent with this. Based on published lesion studies, deficits in the object-in-place task but not the object location task would indicate defects in the PFC not HPC, since PFC is believed to mediate memory for object location (Òobject displacementÓ), whereas HPC integrates information as to object identity and the temporal order of object presentation with HPC lesioned animals being impaired on object location task [16], [17], [21]. However, further to the above discussion, there is also increasing recognition that the cerebellum itself has a direct role in cognition [22] and it is possible that some of the phenotype results directly from cerebellar abnormalities. Further investigation should also allow a detailed analysis of which specific brain regions mediate mild/moderate cognitive impairment in humans.

**Figure 7:**
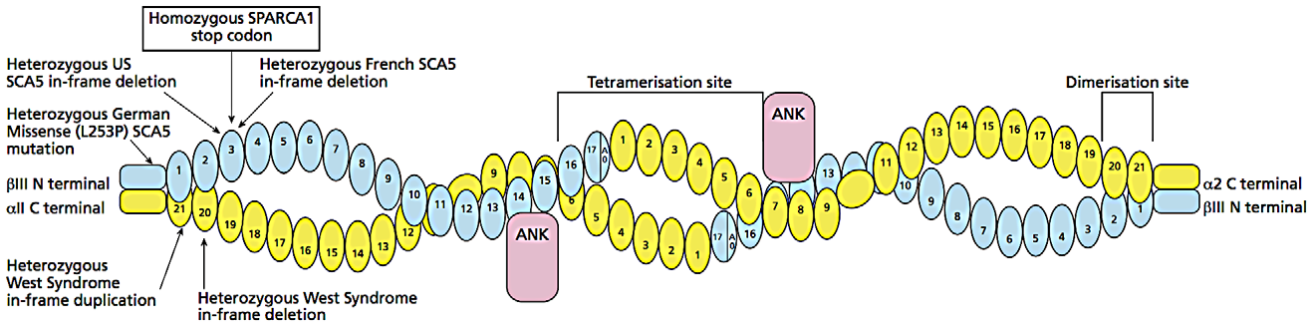
Diagram of β-III spectrin/α-II spectrin tetramer. This is composed of 2 β-III spectrin and 2 α-II spectrin molecules and the location of the homozygous stop codon C627X in *SPTBN2* causing SPARCA1 relative to dominant mutations in *SPTBN2* and *SPTAN1*. Mutations are only shown in one of the two molecules. Loss or truncation of β-III is likely to prevent formation of normal tetramers. The glutamate transporter, EAAT4, binds near the C terminus of β-III.

The data demonstrate that our β-III spectrin knockout mouse [13] is an excellent model for the novel recessive disorder we have identified and will allow further molecular analysis of β-III spectrin, in addition to the morphological and behavioural analysis. β-III spectrin is known to be expressed widely throughout the brain, kidney, liver and testes and to be associated with the Golgi and other cytoplasmic vesicles [23], but the mechanisms by which mutations lead to impaired brain development are unknown. The premature stop codon C627X identified in our family is predicted to result in truncation of β-III spectrin near the end of the 3^rd^ spectrin repeat (Figure 7). This truncated protein would be unable to form tetramers with α-II spectrin, nor be able to bind to EAAT4 or ankyrin, but it is possible that there is nonsense mediated decay and loss of the entire protein. Since *SPTBN2* is expressed at only very low levels in peripheral blood, further *in vitro* expression studies will be required to determine this. However, it is most likely that β-III spectrin is absent in the brain of the human subjects and this has resulted in neuronal dysfunction in widespread brain regions, notably cerebellum and prefrontal cortex. Future studies will investigate other brain regions such as striatum and perirhinal cortex as well.

Our findings also provide insights into the mechanism of molecular dominance in SCA5: the heterozygous carrier parents of the C627X stop codon in the SPARCA1 family are neurologically normal despite carrying a stop codon which in the homozygous state is a recessive loss of function mutation. Therefore haploinsufficiency is highly unlikely to be the mechanism underlying SCA5 and this lends considerable weight to the body of experimental evidence suggesting that SCA5 results from a dominant negative effect, possibly by interfering with normal binding to ARP1 [13], [14], [24].

One difference between the human and mouse model is that the mouse shows progressive motor deficits in addition to progressive Purkinje cell loss whereas there is no evidence of clinical progression in the patients at the moment despite one of our subjects having progressive cerebellar atrophy on imaging. This lack of clinical progression and discordance between the clinical and imaging findings could suggest that there is significant plasticity within the human cerebellum, although we cannot exclude the possibility that slow clinical progression will occur with time.

The phenotypic spectrum of neuronal spectrinopathies now appears to be very wide. In SCA5, the ataxia is generally a pure adult-onset ataxia whereas recessive mutations in *SPTBN2* cause SPARCA1, a more severe childhood ataxia with cognitive impairment. In West Syndrome, associated with *SPTAN1* mutations, the patients have epilepsy, profound developmental delay and in addition have shortening of the corpus callosum and cerebellar vermis atrophy. Only one of our patients, V3, had shortening of the corpus callosum and it is tempting to speculate that this additional feature may be part of the SPARCA1 phenotype, although there are no signs of hypoplasia in the β-III spectrin knockout mice. It also may be that this feature is caused by another gene mutation or a genetic modifier and to clarify this additional cases will need to be identified. Overall, our data suggest that region specific expression of spectrin subunits is important in prenatal brain development and further work is required to define their temporal and spatial contribution.

Our data also suggest the possible and testable hypothesis that the phenotype in neuronal spectrinopathies relates in part to the total amount of functional spectrin tetramers: in SCA5, all α-II/β-II tetramers are normal and functional but α-II/β-III tetramers will contain mutant β-III spectrin which likely have a dominant negative action and may not be fully functional; in SPARCA1, a recessive disorder, there is complete loss of the tetramerisation site of β-III spectrin so there will be normal α-II/β-II tetramers but no functional α-II/β-III tetramers, whereas the heterozygotes who are effectively “haploinsufficient” have enough α-II/β-III tetramer to be clinically normal; in West Syndrome, caused by in-frame dominant *SPTAN1* mutations [15], the majority of both α-II/β-II and α-II/β-III tetramers are abnormal resulting in the most severe of the disorders to be described so far (Figure S2). This model would suggest that homozygous loss of function α-II spectrin mutations might be more severe or lethal and a very recent report of an α-II knockout mouse supports this and it will be important to identify the equivalent human disorder [25]. There may be other disorders associated with human disease: dominant negative or recessive mutations in β-II and proteins interacting with brain spectrins may also have similar phenotypes. For example, a mouse knockout model of Ankyrin G, was reported to cause Purkinje cell degeneration [26] but a human phenotype has not yet been found. In addition, seizures are described in *SPTAN1* mutations [15] and another β-III spectrin knockout [24] and it will be important to search for spectrin mutations in epilepsy patients.

In conclusion, the identification of recessive mutations in β-III spectrin provides evidence that the cell-specific repertoire of spectrin subunits underlies a novel group of disorders, the neuronal spectrinopathies, including SCA5, a dominant form of West Syndrome and SPARCA1. It is likely that other human disorders are caused by mutations in neuronal spectrins and searches for these are in progress. We also demonstrate the power of analysing complex phenotypes in consanguineous families by using whole genome sequencing, which was critical in establishing that both the ataxia and the cognitive impairment were caused by the same mutation and illustrate how the use of genome sequencing, even in single human families, can help provide mechanistic insights into disease.

## Materials and Methods

### Ethics on study participants and animal analysis

Our institutional ethics committee approved the study on human participants and specific consent was obtained to include whole genome analysis. All procedures involving analysis of mutant mice were carried out according to the United Kingdom Animals (Scientific Procedures) Act (1986) and other Home Office regulations under specific pathogen-free conditions.

### Targeted resequencing and analysis

The exonic sequences of 129 genes known or suspected to be associated with ataxia were selected for targeted capture (Table S1) and 120-mer baits with 2X tiling designed using the Agilent eArray design tool. The total size of the targeted region amounted to 605.8 kb. Multiplex sequencing was performed on the Illumina GAII with 51 bp paired-end reads. A total of 5,046,154 reads were generated for patient V3 and aligned to the human reference genome (GRCh37/hg19) with STAMPY [27] About 60% of the reads mapped to the target region, providing a mean depth coverage of 218.4× with 89.8% of target bases covered at 30× or more. Single nucleotide variants (SNVs) and indels were called respectively with SAMTOOLS [28] and DINDEL [29]. Variants were annotated with respect to gene and transcripts using the Ensembl database (release 62, Apr 2011 [30]) by means of the associated Variant Effect Predictor tool.

### Confirmation of variants using Sanger sequencing

Results were confirmed using Sanger Dideoxy Sequencing with the following primers across exon 14 of *SPTBN2*: Forward: CTACCTCTGCTGCACGACCT; Reverse: AGGGAGGGAAGTCCAAGAGA. Genomic DNA was amplified with Taq Polymerase (Roche) and PCR products were used as templates for sequencing with BigDye Terminator reagents (Life Technologies) on a 3730xl DNA Sequencing Analyzer (Life Technologies). The sequence traces were aligned to the gene-specific reference sequence (NCBI build 37) with Sequencher 4.10.1 (Gene Codes).

### SNP array genotyping and homozygosity mapping

Genotyping was performed using the Illumina HumanCytoSNP-12v1 BeadChip, containing nearly 300,000 genetic markers. Hybridization to the chip was performed according to manufacturer's protocols found on registration at http://www.illumina.com/support/array/array_kits/humancyto-snp-12_v2-1_dna_analysis_kit/documentation.ilmn. In brief, patient DNA was denatured, amplified and enzymatically fragmented and then hybridized onto CytoSNP-12 BeadChips by rocking in an Illumina hybridization oven at 48°C for 16–24 hrs. The BeadChips were washed according to the Illumina Inc. protocol and the hybridized DNA detected by primer extension with labelled nucleotides followed by detection using fluorescent antibodies. The data were processed using Illumina's GenomeStudioV2009.2.

As SNP coordinates in the chip were reported with respect to human genome build 36, we downloaded the corresponding coordinates for build 37 from the website http://www.well.ox.ac.uk/~wrayner/strand/, cross-checking them using the USCS Genome Browser liftOver utility (http://genome.ucsc.edu/cgi-bin/hgLiftOver) and the dbSNP database (Build 135 [31]). We filtered out ∼18,000 markers which could not be mapped unambiguously to build 37 of the human genome. We further excluded SNPS with missing calls in one or more samples, thus reducing the number of markers to 271,208.

PLINK v1.07 (http://pngu.mgh.harvard.edu/purcell/plink/ [32]) was used to identify regions of homozygosity (ROH) shared by V1, V2 and V3 and not present in either IV3 or IV4. For V1, V2 and V3, we applied relaxed parameters in order to include all potential ROH, resulting in potential false positives but minimizing false negatives. We defined a homozygous region as a run of (at least) 50 homozygous SNPS spanning more than 500 kb, allowing for some heterozygous calls within it. Shared ROH were identified from overlapping and allele matching segments. Further details of the algorithm are provided on the PLINKwebsite. We used the options: –homozyg –homozyg-group –homozyg-window-kb 500 –homozyg-window-snp 50 –homozyg-snp 50 –homozyg-kb 500. All other parameters were left at default values. ROH were then identified in IV3 and IV4. In this case very stringent criteria were applied to confidently include only true ROH and avoid false positives. We defined a homozygous region as an uninterrupted run of (at least) 500 homozygous SNP's spanning more than 5 Mb. In IV3 we identified 8 ROH on autosomes totalling 78 Mb (the largest ROH was 18.4 Mb); in IV4 we identified 2 large ROH on chromosome 11 present also in V1, V2 and V3 (Table 2 and Figure 3). These regions were excluded in the search for pathogenic variants as both IV3 and IV4 are unaffected. As a result, the search was restricted to 20 regions totalling 17.1 Mb, among which the ROH harbouring *SPTBN2* was the largest.

### Whole-genome sequencing

#### Data generation

Whole-genome sequencing of patient V2 was carried out as part of the WGS500 project, a collaboration between the University of Oxford and Illumina to sequence 500 whole genomes of clinical relevance. (http://investor.illumina.com/phoenix.zhtml?c=121127&p=irol-newsArticle&ID=1592299). At time of writing 213 genomes have been completed and have been grouped and organised in the WGS500 Data Freeze 3 (February 2012).

#### Library preparation and sequencing

Samples were quantified using the High Sensitivity Qubit system (Invitrogen) and sample integrity was assessed using 1% E-Gel EX (Invitrogen). 2 ug* of DNA were fragmented using the Covaris S2 system. Libraries were constructed using the NEBNext DNA Sample Prep Master Mix Set 1 Kit (NEB) with minor modifications. Ligation of adapters was performed using 6 µl of Illumina Adapters (Multiplexing Sample Preparation Oliogonucleotide Kit). Ligated libraries were size selected using 2% E-Gel EX (Invitrogen) and the distribution of fragments in the purified fraction was determined using Tapestation 1DK system (Agilent/Lab901). Each library was PCR enriched with 25 µM each of the following custom primers:Multiplex PCR primer 1.0: 5′- -3′ Index primer: 5′ CAAGCAGAAGACGGCATACGAGAT[INDEX]CAGTGACTGGAGTTCAGACGTGTGCTCTTCCGATCT-3′. Indexes were 8 bp long and part of an indexing system developed in-house.

Four independent PCR reactions per sample were prepared using 25% volume of the pre-PCR library each. After 8 cycles of PCR (cycling conditions as per Illumina recommendations) the four reactions were pooled and purified with AmpureXp beads. The final size distribution was determined using a Tapestation 1DK system (Agilent/Lab901). The concentration of each library was determined by Real-time PCR using the Agilent qPCR Library Quantification Kit and a MX3005P instrument (Agilent).

Whole Genome Sequencing was performed on the Illumina HiSeq2000 as 100 bp paired end reads, using v3 clustering and sequencing chemistry. A PhiX control was spiked into the library. We ran 2 lanes of the original library at 21.5 and 23 pM. Then, to “top up” to the required coverage, we ran the library in a multiplex of 16 over 5 lanes at 18 and 18.5 pM

#### Data analysis

WGS reads were mapped to the human reference genome (GRCh37d5/hg19) using STAMPY [27] and duplicate reads removed using Picard (http://www.picard.sourceforge.net/). After duplicate reads removal, the mean coverage across the genome was 25.6× with 90.4% of bases covered at 15× or more. The mean coverage over the 17.1 Mb ROH identified by SNP analysis was 25.9× with 93.4% of bases covered at 15× or more. Coverage was calculated with custom scripts and the BEDTOOLS package [33]. Identification of variant sites and alleles was performed with Platypus (written by Andrew Rimmer, Ian Mathieson, Gerton Lunter and Gil McVean: http://www.well.ox.ac.uk/platypus), which can detect SNPs and short (<50 bp) indels. Briefly, Platypus works by re-aligning reads by putative haplotypes obtained from combining candidate variants, and uses a statistical algorithm to identify the haplotype(s) that best explain the read data, and infer variants and their frequencies.

First, poorly or ambiguously mapped reads are filtered from the data. Platpyus requires a minimum mapping quality of 20, which equates to a nominal 1/100 chance of the read being incorrectly mapped. Reads with large numbers of low quality base-calls (>20 bases with quality <10) are also removed. This filtering helps to remove spurious variant candidates caused by poor quality data or reads mapped to difficult regions (e.g. long homopolymers or tandem repeats).

Variant candidates are considered by Platypus if they are seen at least twice in good quality reads. For SNPs, the variant base must be seen at least twice with base-quality > = 20. Indel candidates are left-normalised, i.e. the inserted/deleted sequence is reported in the left-most position possible.

Platypus then looks in ∼100–200 base windows across the genome, and creates haplotype candidates, based on the list of variants in each window. Each haplotype may contain several variants. A statistical algorithm is used to infer the frequency of each haplotype in the data provided; this algorithm works by re-aligning all the reads to each of the haplotypes, and uses expectation-maximization to estimate haplotype frequencies, and compute a likelihood for each haplotype. Platypus uses these inferred frequencies and the likelihoods to compute a probability for each variant candidate segregating in the data. These probabilities are reported in the final output as a VCF file.

Finally the variants are filtered, to reduce the false-positive rate. First, variants are only called if they have been assigned a sufficiently high posterior probability (the threshold used by Platypus is a phred score of 5). Additional filters are used to remove variants called in low quality reads, or where the variant is only seen on the forward or reverse strand.

We compared the data obtained by the SNP array for V2 with the WGS of V2 and found that 99.85% of the calls were identical, confirming the accuracy of the WGS.

WGS500 Data Freeze 3 (February 2012) includes 213 individual samples. The variant calling was performed as a two step procedure. Initially, variants were called independently for each individual WGS500 sample. The variants from all normal (non-tumour) samples were then merged to generate a union set, containing 26,952,978 unique entries. The second step involved running Platypus on each sample using the variants in the union set as candidates (i.e. as priors). For each variant, the number of occurrences as heterozygous and homozygous in the union set was recorded.

The variants were then processed with a functional annotation pipeline based on the ANNOVAR software package (version of October 2011 [34]). The following ANNOVAR databases (with respect to human genome hg19) were used: RefSeq gene models; dbSNP (Build 132); 1000 genomes allelic frequencies (November 2011); UCSC segment duplication scores; UCSC 46 species conservation scores. Candidate variants were annotated with predictions of functional importance from SIFT [35], PolyPhen2 [36], PhyloP [37] and GERP [38]. We screened known associations to diseases with OMIM (http://www.omim.org/), HGMD Professional (http://www.hgmd.org/) and GeneCards (http://www.genecards.org/).

### Neuropsychological assessments in family

Screening of cognitive function was undertaken using the Wechsler Abbreviated Intelligence Scale (WASI).

### Neuronal cell imaging

For immunostaining and histological analysis brains from wild type and β-III spectrin knockout animals were removed and immersion-fixed with either 1 or 4% paraformaldehyde in 0.1 M sodium phosphate buffer, pH 7.4 overnight at 4°C and cryoprotected in 0.1 M sodium phosphate buffer (pH 7.4) containing 30% sucrose. Tissue was embedded in OCT then 16 µm-thick sections cut and mounted onto poly-L-lysine coated slides. Primary antibodies used were mouse anti-MAP2 (Sigma), rabbit anti-tau (DAKO) and rat anti-myelin basic protein (AbD Serotec). Secondary antibodies were cyanine 3 (Cy3)-conjugated goat anti-mouse IgG (Jackson laboratories), fluorescein isothiocyanate (FITC)-conjugated goat anti-rabbit IgG (Cappel) and Alexa Fluor 488 –conjugated donkey anti-rat (Invitrogen). For Golgi impregnation brains were removed and immersion-fixed with 4% paraformaldehyde in 0.1 M sodium phosphate buffer, pH 7.4 overnight at 4°C and processed as described previously [39]. For cell filling animals were deeply anesthetized with isofluorane and sacrificed by transcardial perfusion with 4% paraformaldehyde in 0.1 mM phosphate buffer, pH 7.4. Brains were dissected and postfixed in 1% paraformaldehyde overnight at 4°C. Coronal sections were cut (250 µm-thick) and individual neurons in layer 2/3 of the prefrontal cortex were visualized with a 20× immersion objective and injected with 0.2 mM Lucifer Yellow (Sigma) and 0.02 mM Alexa FluorAR 568 hydrazide (Invitrogen). Slices were post-fixed and 4% paraformaldehyde overnight at 4°C and wet-mounted with Vectashield onto 0.13 mm thick borosilicate glass and neurons imaged using the Alexa 568 dye. All images were captured using a Zeiss inverted LSM510 confocal scanning laser microscope and serial stacks used for three-dimensional reconstruction of dendritic arbors using NeuronStudio software (CNIC).

### Behaviour tasks in mice

Animals were handled for 1 week and then habituated to the arena (40 cm×40 cm×40 cm) for 5 d before testing. All tests involved a 5 min sample phase followed by a 5 min test phase after a delay of 5 min. Exploratory behaviour was recorded via a WebCam positioned above the testing arena and two researchers blind to genotype scored the investigation of each sample using ANY-maze software (Stoelting). As described previously [16], [21] for the novel object preference tasks one object from the sample phase was replaced with a novel object in the test phase; the object-in-place task comprised switching the location of two familiar objects in the test phase; and for the object location task position of one familiar object was changed (Figure 6A–6D). Duplicate copies of familiar objects were used in the test phases to remove any chance of olfactory cues being present. Discrimination ratios were calculated as the time spent exploring the novel or location switched object(s) divided by the total time spent exploring all objects.

### Statistical analysis of mouse studies

Statistical analysis was performed using Student's *t-test*, two sample assuming unequal variance, apart from analysis of filled pyramidal cells where a two-way ANOVA was used.

## Supporting Information

Figure S1 Normal axonal immunostaining in β-III spectrin knockout mice. A. Coronal and sagittal cortical sections from 8-week old WT (+/+) and β-III spectrin knockout mice (−/−) immunostained for tau (Bar, 20 µm). B. Coronal sections of cortex and striatum (low and high magnification) stained for MBP (Bar, 20 µm).
(TIF)

Figure S2 Possible disease mechanism of impaired spectin tetramer formation. α-II shown in yellow, β-II in dark blue, β-III in light blue. Normal tetramers are shown in Black and the mutant dominant tetramers in red bold text and mutant recessive (loss of function) tetramers in red italic text. In the normal there are 4 possible comibinations of either α-II/β-II or α-II/β-III. In the SPARCA1 heterozygous carriers tetramers containing β-II are all normal, but ¾ of the tetramers containing β-III are either absent (if nonsense mediated decay is present) or truncated. These patients are clinically normal illustrating that the tetramers with mutant β-III do not have a dominant effect nor is there haploinsufficiency. In SCA5 the same total number of spectrin tetramers are present as in the heterozygous carriers of the SPARCA1 mutation, but they are clinically affected and the mutation therefore must have a dominant negative effect, rather than be caused by haploinsufficiency. In SPARCA1 all α-II/β-III are non-functional, and in West syndrome, both α-II/β-II and α-II/β-III tetramers are affected resulting in a more severe phenotype.
(TIF)

Table S1 Ataxia genes screened by targeted next generation sequencing. The panel included 117 genes known to cause ataxia in humans, in animal models or were considered likely candidates based on function.
(DOC)

Table S2 Compound Heterozygous variants identified in V2. Compound heterozygous variants identified in V2 filtered as detailed in Material and Methods. a = Wellcome Trust Centre for Human Genetics Whole Genome Sequence data, Freeze 3. b = Exome Variant Server. c = SIFT Probability of being pathogenic; 0 = highest; 1 = lowest. d = Polyphen2 Probability of being pathogenic: 0 = lowest; 1 = highest. e = PhyloP, measures conservation at individual columns of nucleotides. f = PhastCons, is a hidden Markov model-based method that estimates the probability that each nucleotide belongs to a conserved element. g = GERP, Genomic Evolutionary Rate Profiling (GERP) (35 species alignment) conservation score.
(DOC)
