# Supplementary figures and images for: Recessive Mutations in SPTBN2 Implicate β-III Spectrin in Both Cognitive and Motor Development
Source: PLoS Genet. 2012 Dec 6;8(12):e1003074. doi: 10.1371/journal.pgen.1003074 (PMC3516553; doi:10.1371/journal.pgen.1003074)

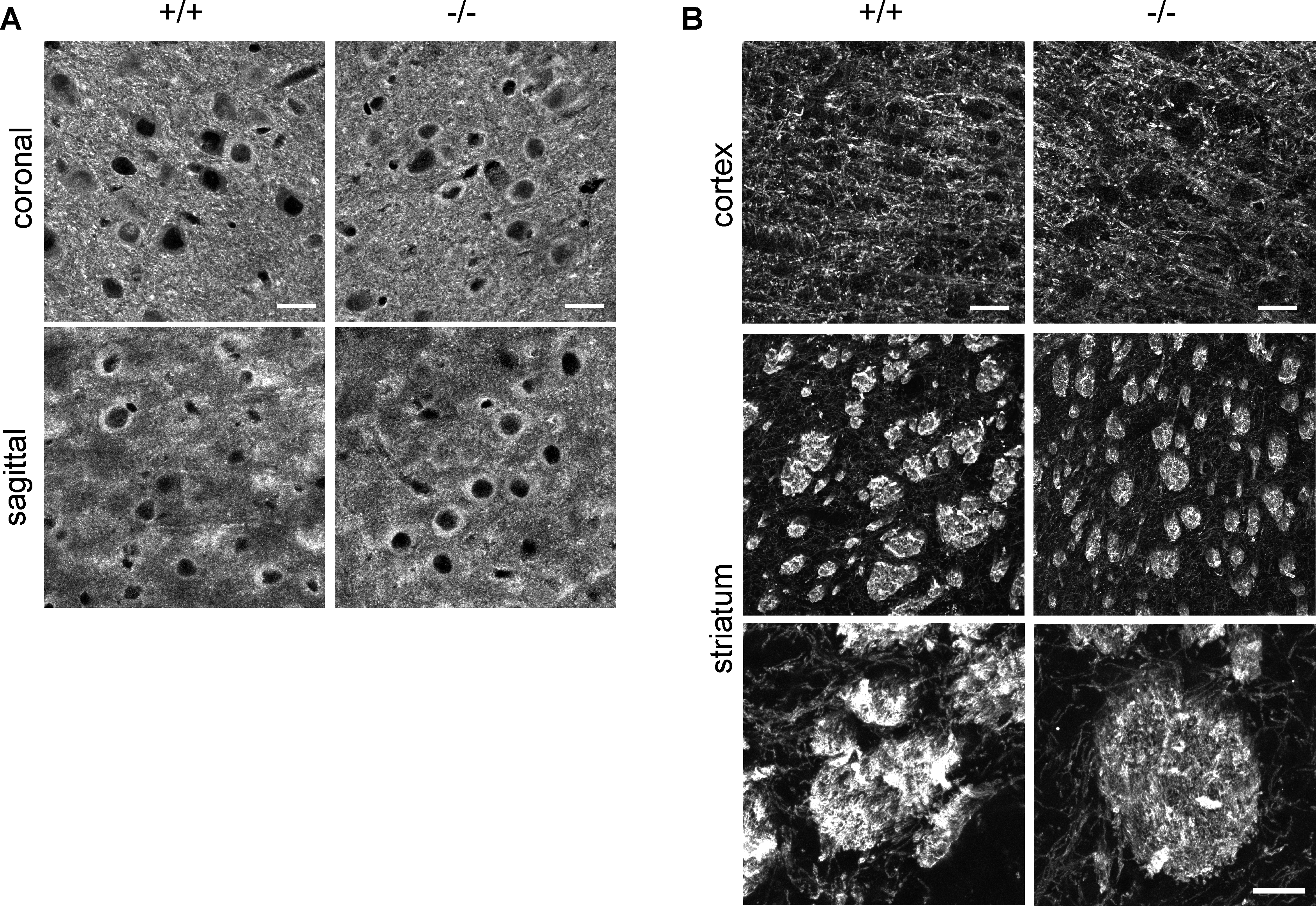

Supplement: Figure S1 — Normal axonal immunostaining in β-III spectrin knockout mice. A. Coronal and sagittal cortical sections from 8-week old WT (+/+) and β-III spectrin knockout mice (−/−) immunostained for tau (Bar, 20 µm). B. Coronal sections of cortex and striatum (low and high magnification) stained for MBP (Bar, 20 µm). (TIF) [file pgen.1003074.s001.tif]

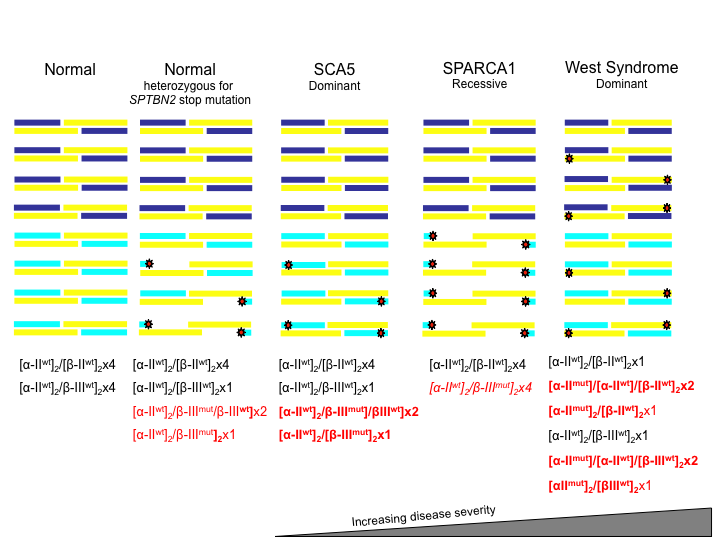

Supplement: Figure S2 — Possible disease mechanism of impaired spectin tetramer formation. α-II shown in yellow, β-II in dark blue, β-III in light blue. Normal tetramers are shown in Black and the mutant dominant tetramers in red bold text and mutant recessive (loss of function) tetramers in red italic text. In the normal there are 4 possible comibinations of either α-II/β-II or α-II/β-III. In the SPARCA1 heterozygous carriers tetramers containing β-II are all normal, but ¾ of the tetramers containing β-III are either absent (if nonsense mediated decay is present) or truncated. These patients are clinically normal illustrating that the tetramers with mutant β-III do not have a dominant effect nor is there haploinsufficiency. In SCA5 the same total number of spectrin tetramers are present as in the heterozygous carriers of the SPARCA1 mutation, but they are clinically affected and the mutation therefore must have a dominant negative effect, rather than be caused by haploinsufficiency. In SPARCA1 all α-II/β-III are non-functional, and in West syndrome, both α-II/β-II and α-II/β-III tetramers are affected resulting in a more severe phenotype. (TIF) [file pgen.1003074.s002.tif]
