# Supplementary material for: Recessive Mutations in SPTBN2 Implicate β-III Spectrin in Both Cognitive and Motor Development
Source: PLoS Genet. 2012 Dec 6;8(12):e1003074. doi: 10.1371/journal.pgen.1003074 (PMC3516553; doi:10.1371/journal.pgen.1003074)
Supplement: Table S1 — Ataxia genes screened by targeted next generation sequencing. The panel included 117 genes known to cause ataxia in humans, in animal models or were considered likely candidates based on function. (DOC) [file pgen.1003074.s003.doc]

| A2BP1 | CHD6 | KCNJ6 | SCN1A |
| --- | --- | --- | --- |
| AAAS | DARS | KCNV3 | SETX |
| AARS | DARS2 | KIAA0226 | SIL1 |
| AARS2 | DNAJC19 | KLHL1 | SLC12A6 |
| ABCB7 | DST | LARS | SLC1A3 |
| AFF1 | EARS2 | LARS2 | SLC6A19 |
| AFG3L2 | EEF2 | MARS | SNAP25 |
| AGTPBP1 | EN1 | MARS2 | SPTBN2 |
| AHI1 | EN2 | MRE11A | SYNE1 |
| AIFM1 | EPRS | MYO5A | SYNE2 |
| APTX | FARS2 | NARS | TARS |
| ARL13B | FARSA | NARS2 | TARS2 |
| ATCAY | FARSB | NPHP1 | TBP |
| ATM | FGF14 | OFD1 | TDP1 |
| ATN1 | FXN | PARS2 | TINF2 |
| ATP2B2 | GAN | PLEKHG4 | TMEM216 |
| ATXN1 | GARS | PPP2R2B | TMEM67 |
| ATXN10 | GRID2 | PRKCG | TRPC3 |
| ATXN2 | GRM1 | PRPS1 | TTBK2 |
| ATXN3 | HARS | PSAP | TTPA |
| ATXN7 | HARS2 | QARS | USP14 |
| ATXN8OS | HERC1 | RAB3A | VARS |
| BEAN1 | IARS | RARS | VARS2 |
| CABC1 | IARS2 | RARS2 | VLDLR |
| CACNA1A | INPP5E | RELN | WARS |
| CACNB4 | ITPR1 | RORA | WARS2 |
| CARS | Joubert | RPGRIP1L | YARS |
| CARS2 | KARS | SACS | YARS2 |
| CC2D2A | KCNA1 | SARS |  |
| CEP290 | KCNJ10 | SARS2 |  |
