# Supplementary material for: Recessive Mutations in SPTBN2 Implicate β-III Spectrin in Both Cognitive and Motor Development
Source: PLoS Genet. 2012 Dec 6;8(12):e1003074. doi: 10.1371/journal.pgen.1003074 (PMC3516553; doi:10.1371/journal.pgen.1003074)
Supplement: Table S2 — Compound Heterozygous variants identified in V2. Compound heterozygous variants identified in V2 filtered as detailed in Material and Methods. a = Wellcome Trust Centre for Human Genetics Whole Genome Sequence data, Freeze 3. b = Exome Variant Server. c = SIFT Probability of being pathogenic; 0 = highest; 1 = lowest. d = Polyphen2 Probability of being pathogenic: 0 = lowest; 1 = highest. e = PhyloP, measures conservation at individual columns of nucleotides. f = PhastCons, is a hidden Markov model-based method that estimates the probability that each nucleotide belongs to a conserved element. g = GERP, Genomic Evolutionary Rate Profiling (GERP) (35 species alignment) conservation score. (DOC) [file pgen.1003074.s004.doc]

| Gene | Function if known/Disease association | AA change | WGS500 (Het, including this sample)^a^ | EVS^b^ | SIFT^c^ | PolyPhen2^d^ | PhyloP^e^ | Phast Cons^f^ | GERP^g^ | Segregation |
| --- | --- | --- | --- | --- | --- | --- | --- | --- | --- | --- |
| CAPN14 | calcium-activated neutral proteinase 14; no known disease association | I632V | 1 | 0 | 0.9 | 0 | -1.195 | 0.002 | -4.94 | V1, V2, V3 |
|  |  | V399M | 1 | 0 | 0.18 | 0.797 | -0.048 | 0.001 | 0.972 | V2 only |
| CHD7 | CHARGE syndrome, dominant congenital malformation disorder | R1632C | 1 | 0 | 0 | 0.91 | 2.808 | 0.994 | 5.63 | V2 only |
|  |  | E1897K | 1 | 1 | 0.22 | 0.119 | 2.689 | 1 | 5.38 | Not done |
| COL3A1 | Ehlers-Danlos Syndrome type IV, severe abnormality of collagen | P668T | 1 | 0 | 0.02 | 0.851 | 2.805 | 0.982 | 5.93 | Not done |
|  |  | A908G | 1 | 0 | 0.03 | 0.056 | 2.59 | 0.095 | 5.5 | Not present in sib or cousin |
| CROCC | Maintenance of ciliary rootlet in photoreceptors, mutations cause photoreceptor degeneration in mice | R1437L | 1 | 0 | 0.11 | 0.186 | 0.333 | 0.324 | 1.79 | Not done |
|  |  | V1864A | 1 | 0 | 0.13 | 0.093 | -0.183 | 0.975 | -1.59 | V2 and V3 only |
| CTNNAL1 | alpha catulin, function unknown | M716R | 1 | 0 | 0.1 | 0 | 1.053 | 1 | 3.44 | V1 and V2 only |
|  |  | N91T | 1 | 0 | 0.02 | 0.061 | 2.326 | 1 | 6.07 | V1 and V2 only |
| EPPK1 | Epiplakin, which was recently identified as an autoantigen in serum from a patient with a subepidermal blistering disease | V2291M | 1 | 4 | 0.02 | 0.889 | 0.48 | 1 | 2.79 | Not done |
|  |  | T120A | 1 | 0 | 0.29 | 0.111 | 0.747 | 0.487 | 3.28 | V2 only |
| FER1L6 | Gene involved in folliculogenesis or male fertility in homologs of model organisms | V970I | 1 | 2 | 0.57 | 0 | -3.685 | 0 | -11.3 | Both variants in all affecteds, plus both in IV3, therefore variants *in cis* |
|  |  | P1689fs | 1 | 0 | N/A | N/A | N/A | N/A | N/A |  |
| OBSCN | Obscurin localized at the M-line of myocytes from heart, no known disease association | E2275K | 1 | 0 | 0.11 | 0.542 | 0.457 | 0.048 | 2.39 | Not done |
|  |  | R7508H | 1 | 0 | 0.29 | 0.019 | -0.035 | 0.093 | 1.14 | V2 only |
| SSPO | No predicted protein product | N/A | 1 | 0 | N/A | N/A | N/A | N/A | N/A | V1 and V2 only |
|  |  | N/A | 2 | 0 | N/A | N/A | N/A | N/A | N/A | Not done |
| TRPM5 | TRPM5 is a cation channel that it is essential for transduction of bitter, sweet and umami tastes | L939P | 1 | 0 | 0.04 | 0.718 | 0.407 | 0.026 | 2.42 | Not done |
|  |  | P761L | 1 | 0 | 0.01 | 0.996 | 0.921 | 0.019 | 3.36 | V2 only |
| XIRP1 | Xin-actin-binding repeat-containing protein1, marker for wounded skeletal muscle cells, animals deficient for cardiac XIRP are still viable | 1192_1194del | 1 | 0 | N/A | N/A | N/A | N/A | N/A | Not present in cousin |
|  |  | S953T | 1 | 0 | 0.03 | 0.01 | 0.648 | 0.114 | 1.92 | Not present in cousin |
| ZDBF2 | Zinc finger gene; no known disease association, paternally expressed imprinted gene | G2278fs | 1 | 0 | N/A | N/A | N/A | N/A | N/A | Not present in cousin |
|  |  | 2279_2279del | 1 | 0 | N/A | N/A | N/A | N/A | N/A | V1 and V2 only |
| ZNF792 | Zinc finger gene; no known disease association | A614T | 2 | 2 | 0.00 | 0.002 | 0.157 | 0 | 0.355 | V2 only |
|  |  | S605T | 2 | 1 | 0.38 | 0.001 | -0.75 | 0.006 | -3.6 | V2 only |
